# Emotional intelligence as a conduit for improved occupational health safety environment in the oil and gas sector

**DOI:** 10.1038/s41598-023-46886-3

**Published:** 2023-11-11

**Authors:** Nkrumah Nana Kwame Edmund, Liu Suxia, Larnyo Ebenezer, Arielle Doris Tetgoum Kachie

**Affiliations:** 1 School of Management and Safety Engineering, Jiangsu University, Zhenjiang, China; 2 School of Management, Jiangsu University, Zhenjiang, China; 3 Department of Health Policy and Management, School of Management, Jiangsu University, Zhenjiang, China

**Keywords:** Health occupations, Occupational health, Psychology and behaviour

## Abstract

To address the issue of promoting occupational health and safety at the workplace, this study aimed to evaluate the mediating effect of four different dimensional constructs of Emotional Intelligence (EI) on the influence Occupational Health and Safety Management Practices (OHSMP) hold on safety performance and workplace accidents among oil and gas workers. The study is explanatory research that adopted a cross-sectional survey design. Convenience and stratified sampling techniques were used to select 699 respondents from the three major government-owned oil and gas organizations. The multiple standard regression and bootstrapping mediation methods were used for data analysis after subjecting the data to exploratory and confirmatory factor assessments. Results indicated that OHSMP significantly predicts EI, safety performance, and workplace accidents. Again, EI was found to predict safety performance and workplace accidents significantly. Results also indicated that all the construct dimensions for measuring EI significantly explain the relationship between OHSMP and safety performance, as well as the influence of OHSMP on workplace accidents. The theoretical basis for these findings is that workers with high-level EI are likely to cope with occupational health and safety lapses or safety-related challenges at the workplace by participating and complying with the organization’s safety management practices or procedures. Such employees are likely to exhibit safe working behaviors and contribute to improving safety performance in the organization.

## Introduction

The oil and gas industry is undoubtedly one of the most lucrative industries in the world. Its significant contribution to industrialization, economic growth, and, most importantly, poverty reduction in most African nations cannot be underplayed. On the contrary, the industry has been chronicled as one of the most hazardous work environments. All phases of operations in the value chain process, thus, from exploration to development and production, expose employees in the industry to a varying degree of risk. Workers in this industry face hazardous exposures such as hydrocarbon leakages, falling objects, fires, explosions, blowouts, and hydrogen sulfide emissions^1^. The 1988 Piper Alpha disaster, Ocean range oil rig disaster, Enchova Central Platform disaster, Bohai 2 oil rig disaster, Foxborough accident, Seveso disaster, BP Deepwater Horizon Oil Spill disaster, Gunashi oilfield fire outbreak, Texas City refinery explosion, Big Spring Alon refinery, Chevron limited oil rig explosion and the famous BP Deepwater Horizon Oil Spill disaster in 2010 are among major accidents in the oil and gas industry that claimed numerous lives, caused disabilities and resulted in great damage to equipment and properties^2^. The Ghana Health Service and Ministry of Health^3^ reported work-related injuries such as slips and falls, electrical shocks, and burns as common among workers. Additionally, the study by Oppong^4^ confirmed that occupational injuries such as contusions, cuts, and lacerations of the legs, hands, and fingers are mostly suffered by workers in the Ghanaian oil and gas sector.

In the center of these catastrophes, previous studies have identified the lack of safety compliance as a significant predictor of workplace accidents, specifically in the oil and gas sector^5–7^. The yearly assessment of incident data reported by the International Association of Oil and Gas Producers (OGP) as well highlighted safety violations due to the refusal to comply with safety procedures as a common factor causing serious catastrophes and accidents among oil and gas organizations^8–10^. Thus, irrespective of existing health and safety systems and standards, workers' violation of safety rules and procedures is likely to contribute to accidents encountered at work. Accidents can, therefore, not entirely be prevented or controlled through the effective implementation of health and safety systems but can also be attributed to human errors committed at work due to the refusal of employees to comply with safety guidelines and procedures or participate in safety-related activities. Theories such as the Heinrich accident causation theory, Behaviour-Based Safety (BBS), Turner’s model of accident causation, and the Swiss-Cheese model have all associated accident causation with human errors relating to safety at work.

The behaviour of humans is complex and hard to understand. Human actions are influenced by several factors such as culture, ethics, religion, environment, education and knowledge, experience and lessons, exposures, personality, genetics, and most importantly emotions^11,12^. Although each factor is expected to explain the reasons why people will act differently from the expected at work, (i.e., failure to participate and comply with safety guidelines and procedures)^13^, the current study focuses on employee emotions, specifically, on the employees' perception of their EI. While prior research has attempted to link EI to safety outcomes or some aspects of safety management practices or safety behaviors and attitudes (e.g., safety training;^14–16^, this study advances the research issue to rather assess the mediating role of EI in the OHSMP-safety performance nexus which has currently been ignored. Thus, this study aims to evaluate the mediating effect of four different dimensional constructs of EI on the influence OHSMP holds on safety performance and workplace accidents among oil and gas workers.

### OHSM practices and safety performance

Globally, organizations have responsibilities for the protection and improvement of the quality of life of workers, hence the management of risks and hazards at the workplace has become an essential element of organizational operational activities. As Kuusisto^17^ put it, safety is a “reliable control of harm,” whiles health refers to the affirmation of the physical, mental, and social well-being of workers. Irrespective of this proposition, risk and hazard management among organizations, particularly highly risky industries, differ in administration and efficiency. Achieving functioning and systematic OHSM practices as well differs^18,19^.

Several studies have attempted to link safety practices to numerous related safety outcomes, including occupational injuries and accident reduction, safety commitment, safety satisfaction, safety performance, and other related safety events^20–23^. The work of Fan et al.^24^, which reviewed 407 empirical studies on occupational health and safety (OHS) published in 17 leading journals between 1956 and 2019 in both developing and developed countries, revealed that systematic and functioning OHSM systems and practices among organizations save lives and improve the quality of life of workers^25^. Other studies have also shown that a safe working environment predicts positive working behavior, promotes positive risk perception, and improves safety outcomes. According to Abate^26^, an organization that provides safety equipment and educates its employees on its usage is likely to record lower work-related injuries. Occupational health and safety systems can, therefore, be considered by organizations as a significant internal mechanism to control safety outcomes.

The concept of safety performance is a familiar concept in safety management that has gone through tremendous change. However, understanding the level of safety compliance and safety participation as described by Neal and Griffin^27^ continues to be a good indicator for assessing safety performance. As Blakey and Abramowitz^28^ indicated, safety performance is the immediate and contextual attitudes toward preventing and minimizing work-related hazards. In this study, it is anticipated that workers who work in a risky and unsafe environment, such as the oil and gas sector, are likely to be exposed to different hazards, accidents, and work-related injuries. The need for safety compliance and safety participation among workers should therefore be of utmost concern to management and employees. On the other hand, one of the leading indicators to assess the efficiency of the organization’s OHSM practices depends on the level of safety compliance and safety participation among workers.

There is no doubt that the oil and gas sector is among one of the riskiest industries. In Ghana, it is the biggest economic contributor, yet research on this sector is quite limited in scope. The nature and scope of business operations in this industry are quite complex but it is without developed occupational health and safety standards and regulations as compared to sectors in developed countries. Lapses in the effective administration of occupational health and safety policies include the lack of safety policy directions, poor safety infrastructure, lack of funding for safety systems, a large number of unqualified occupational health professionals, inadequate OHS work-related accidents or injuries monitoring mechanism and the unavailability of health and safety data have all been cited as challenges facing Ghanaian industries^20,29–31^. Be as it may be the players in this industry are inevitably faced with numerous hazardous exposures which need utmost commitment and willingness in the effective administration of OHSM practices to improve safety performance among the employees. The study therefore attempted to test the influence of OHSM practices on safety performance among oil and gas workers.

### OHSM practices and emotional intelligence

Unarguably, the concept of health and safety management practices has existed for years, yet organizations continue to record quite a substantial number of occupational accidents and work-related injuries at the workplace. Without doubting the potential and need for organizations to continue investing in effective safety management systems, it is also significant to say that the mere existence and good practice of OHSM may not necessarily promote work safety but rather the interconnection between the worker, the safety management practices and the degree to which they connect their emotions and cognitive ability to the work they do. Basically, organizations adopt safety management system approaches to manage and control their safety functions in an attempt to achieve good safety outcomes and performance excellence. However, in the field of human performance improvement, there exist several cognitive performance tools that can be used to control and reduce the chances of human errors in the workplace. Organizations have also used them to provide mental and social skills that complement a worker’s technical skills to promote safe and efficient task performance^32^. Some previous studies have also criticized most OHSM practices for being too focused on structural elements, whereas organizational and social factors have been subjected to insufficient attention^33,34^.

Individuals are naturally proactive and intuitively rational in their eagerness toward personal growth and improvements and may exhibit psychological needs that are innate, universal, and significant for the maintenance of safety and health. A balanced and effective OHSM practice should therefore be cognitive and emotionally centered. The large workforce of an organization in these industries remains a major factor that drives organizational performance, hence, the importance of sustaining their quality of life in relation to improving their level of EI at the workplace is very necessary. The study, therefore, assumes that an unsafe working environment is likely to distract employees, increase the level of risk perception, reduce concentration, and subsequently affect workers’ EI. On the contrary, an effective safe working environment is expected to promote positive work behavior which may also consequently improve workers’ EI. The main aim here is to diagnose the causal link between OHSM practices and EI among oil and gas workers.

### Emotional intelligence and workplace accidents

Creating a conducive and safe environment for employees is an obligation for every employer, irrespective of the size of the organization. However, the nature of certain industries (e.g., construction, oil, and gas) makes them particularly vulnerable to incidents and accidents even when a lot of investment is sunk into improving occupational health and safety management systems. Human errors are inevitable; however, when it happens, workers should have the ability to handle the situation intelligently. Understanding human errors as part of safety management practices and the continuous engagement of workers in safety activities may likely be one of the critical interventions that can be used to manage workplace accidents, especially those emanating from human errors. EI has mostly been linked to several behavioural traits and has been used to predict different aspects of human behaviour at work^35^.

In addition, during work engagements, there is always a continuous interaction between humans, machines, and organizational policies. In the process of this interaction, accident-related human errors may occur, yet may not necessarily be caused by failure or inefficient safety management systems. This is to say that accident and incident related events at the workplace go beyond improvement in safety management practices but as well the control workers may have to still adhere to safety management practices even within an ever-changing work surrounding.

Employees who engage in safety-related behaviour are mostly connected mentally, emotionally and physically to ensure the safe execution of their tasks without any hindrance. In a highly risky work environment such as the oil and gas sector, the use of emotions to take critical safety-related decisions is not far-fetched. Thus, employee safety engagements and the ability to perceive and control risk at the workplace may be determined by their level of EI. Similarly, EI can act as a personal defence against dealing with accident-related exposures at the workplace. The study of Petitta et al.^35^ found the relationship between cognitive failures and accidents encountered at work to be fully mediated by either positive or negative emotional contagion. Whereas the function of safety management practices is to provide safety at the workplace by controlling and eliminating accident exposures, it is time for organizations to integrate EI into the plan and implementation of safety systems as a control measure for reducing workplace accidents. As a result, understanding how EI is related to safety outcomes such as workplace accidents is critical in this study.

### Emotional intelligence and safety performance

Studies on EI have gotten a lot of attention in management over the years because of their significant implications on worker safety and overall work outcomes^36–38^. Numerous empirical studies have shown that EI improves people's performance at work^36,39^, while other studies have linked EI to particular human traits like understanding one's own and other people's emotions and managing and using those emotions to enhance task and context performance^37,40^. Pena et al.^41^ argued that employees who possess higher EI are more inspirational, vigorous, and enthusiastic and exhibit high concentration and energy towards work. Later, the paradigm shifted to other related dimensions, such as understanding the effect of EI on improving leadership, work engagement, stress management, conflict resolution, improvements in work relationships, and job satisfaction^42–45^.

Empirical evidence related to the EI-safety performance association is still developing. As described by Mayer and Salovey^46^, EI encompasses the individual’s ability to perceive and express emotions, access, and generate emotions to assist thought, understand emotions and emotional meanings, and regulate emotions reflectively to promote both better emotion and thought. The idea of EI, as outlined, is crucial for comprehending and being conscious of one's emotions as well as those of others, which are fundamental in influencing one's behavior and helping one make better cognitive decisions or choices in challenging circumstances. Ifelebuegu et al.^15^, in an attempt to assess the success factors of EI that affect safety performance, concluded that ‘Being aware of one’s own emotions, which means ‘having the aptitude to determine your emotions, thoughts, feelings and physical states’ vastly stimulate safety performance. This succeeded by ‘Being able to rule one’s emotions to facilitate thinking. “Being able to recognise the emotions of others at any given instant in time” showed a significant effect but recorded the lowest influence on safety performance.

Further, correlation analysis indicated that EI success factors of ‘being able to rule one’s own emotions to facilitate thinking, ‘being able to deal with the emotions of others and ‘being able to discuss one’s own emotions accurately’ were found to have a strong significant association with health and safety performance of workers. These findings stress the need for organizations to focus on the management of workers’ ability to ascertain their emotions, thoughts, feelings, and physical states and as well have the capability to rule one’s own emotions to facilitate thinking to improve safety performance or take good safety decisions. Additionally, safety-related decisions, including the level of understanding and compliance with protective regulations for machines and equipment used in the workplace, the ability to take cognizance of a colleague’s inability to make correct decisions in times of emergencies in the workplace and the willingness to participate in meetings pertaining to occupational health and safety measures in the workplace were all found to be strongly determined by EI. Workers’ understanding and perception of risks in the workplace and their willingness to participate in the implementation of risk-reduction action plans in the workplace were also cited to be strongly associated with EI^15^. These findings corroborate with the work of Alsulami et al.^47^ who also indicated that EI plays an important role in improving the safety behaviours of construction workers besides reducing workplace stresses.

According to Guo et al.^48^ unsafe actions by humans are considered one of the major reasons for job-related accidents. Endsley and Robertson^49^ argued that the ability of workers to be aware of what is happening around them at the workplace involves critical appraisal of environmental cues, processing vital safety information, forecasting near-future occurrences, and seeking solutions to control and manage hazardous exposures or emerging risks. That is, safety performance is largely linked to an individual’s self-consciousness which is also directly linked to EI^16,50^. As Petitta et al.^35^ put it, workers with poor cognitive and emotional failures have greater chances of job-related mishaps. In a risky working environment such as the oil and gas sector, where safety performance is of utmost importance, workers can easily get frustrated and discouraged due to the hazardous nature of their work. Again, challenges such as ineffective occupational health and safety management systems coupled with high productivity demands from the organization may trigger negative emotions which could also negatively influence their approach towards safety compliance and participation. Against this backdrop, there is a need to understand EI’s influence on safety performance in the oil and gas sector in order to build a strong theoretical foundation that will encourage managers to incorporate EI related outcomes into work and safety management practices.

### Emotional intelligence as a mediator

The concept of EI is multifaceted and goes beyond the management and control of one’s own emotions, but also how one’s own emotions affect others. As opined by Leung at al.^51^, an accident-prone situation at the workplace may likely raise emotional reactions, hence, the traits or abilities that can regulate the control of undesirable emotional experiences should help employees keep safe at the workplace^52^.

Notably, the causal relationship between occupational health and safety management, safety performance, and workplace accidents has received much attention in previous studies^27,53–56^. Other related studies have as well used different mediating factors such as the workload of employees^57^, safety leadership^58^, safety knowledge^23^, employee involvement^59^, production pressure^60^, burnout^61^, and comprehensibility^62^ to explain the safety-safety performance phenomenon, yet, EI as a mediator has not been currently used to explain this dynamic association. Meanwhile, the concept of EI and its association with safety-related activities, including the understanding of employee safety behaviours and attitudes at the workplace, has gained some ground among researchers^14,15,35,42,63^.

The campaign to enhance safety performance and the promotion of a risk-free or accident-free work environment requires workers to exhibit a high level of self-awareness, self-emotional control and understanding and dealing with the emotions of co-workers, subordinates and superiors. Emotional exhaustion at the workplace for instance has been linked to different adverse health consequences such as depressive and anxious disorders^64,65^, systematic headaches^66^, musculoskeletal disorders^66^, acute or chronic fatigue^67,68^, sleep problems^69,70^, and causing a decline of performance at the workplace. Higher rates of job absenteeism and consistent turnover rates of workers have also been named as an effect of emotional exhaustion^71,72^. The study by Sunindijo and Zou^38^ in an attempt to investigate the influence of EI in typical safety-critical work contexts such as construction settings, revealed that EI facilitates workers in implementing safety management tasks. Besides, Ifelebuegu et al.^15^ have also highlighted how some EI success factors influence safety performance in the workplace.

Even with the tremendous contribution of previous studies on EI, this current study expands the theoretical literature on EI by exploring the mediation role of EI in the influence OHSMP has on safety performance and workplace accidents. As stated earlier, prior studies have not considered EI as a conduit to safety management systems, and safety performance relationships can be determined. Considering the strong ties between EI and occupational safety management as well as EI and safety-related outcomes and behaviours, it is essential to put forward this study to elaborate on the significant role of this relationship.

### Conceptual model

**Figure 1:**
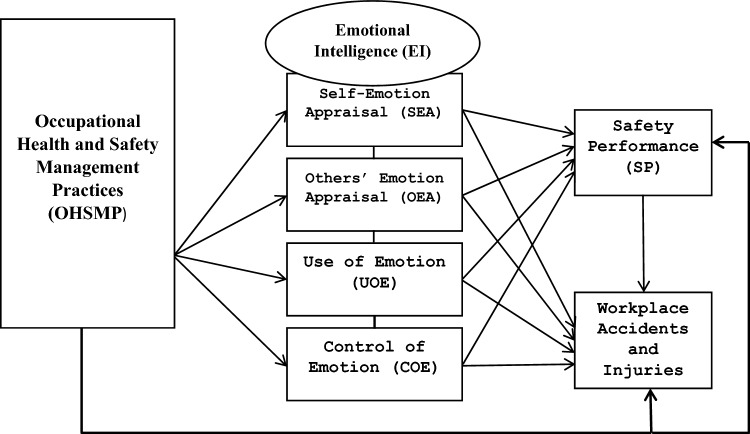
Conceptual model.

When people make good decisions, irrespective of their emotions, they are regarded as emotionally intelligent: Smart emotions make smart people; smart people make smart decisions^73^. Mayer and Salovey^46^; Alkoze^74^ explained EI as the authority to perceive, manage and comprehend emotions. Emotions affect peoples’ attitudes, behaviours, reactions and responses to events around them^44^. Expressing good or negative emotions at work can influence employees' participation and safety compliance, influencing human mistakes and workplace accidents or enhancing organizational safety outcomes. As posited by Khalili^75^, who referred to the daily workplace as an emotion-eliciting environment, there exists a variety of emotional distractions from intrapersonal factors (e.g., personal health and family issues), interpersonal factors (e.g., the relationship with colleagues and supervisors), and other factors (e.g., stressful company or environment) among employees at the workplace. The argument is that such emotional distractions can discompose people’s moods and cause unpleasant feelings or interrupt attitudes and behaviours towards work if not well controlled^76^. Thus, ensuring work safety requires impulse control under the most difficult circumstances. By integrating these ideas, this paper adopted the concept of emotional contagion theory^77^ to develop and test the conceptual model. As advanced by the theory, emotions are socially infectious and spread via workplace contacts; however, workers with higher EI are better able to recognize and manage these emotions, allowing them to operate as emotional conduits. Thus, unpleasant emotions can quickly infiltrate the workplace following workplace accidents. On the other hand, those with high EI are likely to operate as emotional buffers, minimizing the negative emotional effect and supporting their coworkers in an unsafe work environment. The contagion theory is therefore adopted to explain the mediation effect of EI on the influence OHSMP has on safety performance and accidents encountered at the workplace. Thus, the causal relationship between OHSMP and EI, EI and safety performance, EI and workplace accidents, and OHSMP and safety performance was also determined in the study. The objectives of this study are demonstrated graphically in the conceptual model in Fig. 1.

In Fig. 1, ascertaining the influence of OHSMP on each of the four-dimensional constructs on Emotional Intelligence (EI) and determining the influence of each of the constructs of EI on safety performance and workplace accidents is demonstrated. This relationship satisfies the theoretical proponent of the study and the two basic assumptions of performing bootstrapping mediation analysis, as revised by Kenny in 2019. The causal relationship between safety performance and workplace accidents is another interest of the study shown in the framework.

## Materials and methods

### Research approach

An explanatory research approach that uses robust quantitative research methods were adopted for the study. Thus, a cross-sectional approach through the use of research questionnaires was used to collect data from the study participants. The explanatory study was preferred to other research designs because of the need to investigate and clearly define the research hypothesis proposed^78^.

### Study participants and procedures

Participants for the study were drawn from the Ghanaian oil and gas sector, which currently remains one of Ghana's most significant economic contributors. The sector is divided into several jurisdictions, which include the upstream sector, midstream, and downstream sectors. Therefore, the study population focuses on three (3) government-owned oil and gas companies directly involved in all five phases of oil and gas exploration and production.

Convenience sampling and stratified sampling techniques were adopted for the study to select participant groups using Krejcie and Morgan’s 1970 formula table for sampling. With regard to the stratified sampling technique, the population was divided into several groups, and respondents were randomly and proportionally selected from each stratum. This strategy was very significant and appropriate for this study because the respondents’ group for each of the three organizations proportionally varies; hence, there was the need to further divide them into subgroups with similar characteristics before casting the desired sample for the study. It is also essential to state that different job categories exist in the three organizations with varying levels of job risk. Respondents were therefore divided into five (5) strata which include: engineers, maintenance personnel, labourers, technicians, and machine operators. These subgroups of participants were purposively selected following their level of knowledge in safety, working experience, and their direct involvement in risky work-related activities. The convenient sampling technique was finally utilized to cast a proportional sample size from each sub-category.

In all, 750 questionnaires were distributed in three months; however, the analysis for the study was based on the responses of 699 oil and gas workers. The questionnaire administration was a mix of both face-to-face and surveys survey. However, most of the responses were gathered during the emergence of COVID-19. Hence, online video call applications such as WhatsApp, WeChat, skype, and Facetime were utilized to contact participants interested in participating in the study. Questionnaires were sent a week in advance to participants, and follow-up calls were made to solicit responses. With the in-person data collection, participants were met in a group of 5–10 and allowed to complete questionnaires without interruption.

### Research ethics

In adhering to research ethics, the questionnaire was screened by Professor Du's research committee at the School of Management, Jiangsu University. A few comments were made and finally approved by the head of the school’s ethics and research committee. Letters were then officially written to the specific organizations to seek permission for participant data collection. Participation in the study was voluntary. Respondents were further briefed on the objectives and aims of the study before engaging them in the study. Thus, the study followed the Declaration of Helsinki (2013) and included informed consent, ensuring anonymity throughout the process and the option to quit anytime. No respondent in the study was oppressed, punished, or given any form of financial reward to accept or deny participating. These procedures were in accordance with the research guidelines and regulations prescribed by the research and ethics committee of Jiangsu University’s School of Management Science.

### Measures

#### Occupational health and safety management (OHSM) practices construct

What determines an efficient OHSM practice differs in context and dimensions^19,60^ due to several challenges, such as the degree of exposure that differs across organizations, lack of commitment^25^, lack of knowledge^79^, lower financial resources^79,80^, and giving priorities to production and operational activities whiles completely ignoring the safety of workers^18,19^. Be as it may be, one of the most recognized and significant OHSM practices highlighted by previous studies is the degree of management support and commitment to successful safety performance^22,81–84^. The proponent of this assumption has been critically assessed in the Perceived Organization Support for Safety (POSS) theory. Thus, organizations with a strong management commitment toward improving OHSM practices are more likely to improve their safety outcomes (i.e., OHSM-related accidents and injuries) while at the same time may experience positive job outcomes^20,85^.

Irrespective of the lack of consensus on perfect OHSM practices, an effective OHSM represents management responsibilities, practices, procedures, processes, and the investment in resources focusing on occupational, environmental, and social-cultural determinants of health that guide the implementation of accident prevention policies and can maintain the wellbeing of workers over a sustainable period. This study, therefore, focuses on and prioritizes safety guidelines, principles, and practices that are assumed to be built on management safety commitment and leadership, with much focus on safety practices that will improve the health, well-being, and safety behaviours of employees. As described earlier, an accident-prone work environment can trigger employees’ emotions to act negatively against safety and work-related activities. Hence, the study strives to achieve a collective and holistic approach to OHSM practices that focus on more flexible managerial practices that are employee-centered and safety outcome-oriented. Therefore, the OHSM practices investigated in this study, include assessing workers’ perception of organizational safety inspection of hazards, safety policies, procedures and standards, personal protection programs, safety training, and management commitment to safety programs initiated. These five areas of OHSM practices were adopted from the construct of^86,87^. The basic analogy is that the absence of these OHSM practices can affect workers’ emotions and consequently affect safety-related decisions at the workplace. Table 1 below defines each safety management practice.

**Table 1:** OHSM practices.

OHSMP practices	Definition
Organizational safety inspection of hazards	This assesses workers’ perception of the ability of the organization to conduct safety inspections on a daily, weekly, and monthly basis. This includes the safety committee’s ability to strictly conduct monthly audits to review the efficiency and effectiveness of safety programs. Addressing and correcting issues of safety relating to unsafe work conditions and unsafe behaviours among workers as well remain paramount. When hazards exposures are eliminated or controlled at the workplace, workers’ risk perception also reduces, and emotions are expected to be well balanced
Safety policies, procedures and standards	These include safety issues relating to employees’ awareness of safety and health procedures and management's interest in providing information and instruction to employees on relevant legislation and good work practices. When employees are familiar with the safety standards and procedures at the workplace, there is a probability of a reduction in job-related stress and, consequently, an improvement in exhibiting EI at work
Personal protection programs	Assesses employees’ knowledge of the use of the right plants and equipment at the workplace. It reflects workers’ knowledge of the organization’s plant and equipment and familiarity with the safety protocols associated with its usage. Being the last line of defence, there must always be a sufficient and adequate supply of the requisite Personal Protection Equipment (PPE) for all workers in the course of performing their tasks. Conducting regular inspections to replace defective PPEs and follow-up on the quality, standards and effectiveness of the PPE is important. Using the wrong tool or protective gear for the job can cause anxiety or lack of interest which may lead to poor performance. On the other hand, when employees feel safe and protected during working hours, their attention to work may improve and consequently influence their EI
Safety training	Workers need to be trained in hazard communication so that every employee can have vast knowledge about safety compliance, standards and procedures that can improve safe behaviours. Safety training assesses workers’ perceptions of the degree to which they have received sufficient training regarding health and safety issues. When employees are well trained, they will be able to manage safety-related frustrations at the workplace, hence, are likely to exhibit positive encountering accident-related exposures
Management commitment to safety programs	As demonstrated by Perceived Organizations Support for Safety (POSS) theory, management safety commitment denotes management exhibition of commitment towards safety management practices by investing interest in improving employees’ safety-related issues at work. Thus, the provision and implementation of effective health and safety systems should be executed by the top management and supported by the entire organization. In some cases, the structure for safety management systems may exist, yet, the organization does not commit to comply with safety, health, and environmental protection standards, legislation and all the contractual requirements needed to improve safety performance or outcomes. Just as the POSS has been proven to promote positive perceptions among employees, it is also expected that managers’ interest in workers’ safety can be reciprocated with positive emotions towards work

#### Emotional intelligence (EI) construct

EI has been studied extensively, and today there are a variety of measurement instruments available for assessing EI. In this study, EI refers to the power of workers to perceive, manage and understand the personal emotions and emotions of others in relation to their work environment. Thus, the study took into consideration the extent to which workers can intelligently exhibit or manage their emotions and relate to other people’s emotions. Against this context of EI, the construct was measured using the^88^ Scale (WLEIS) scale. The WLEIS was designed for the work context, and it evaluates the assessment and expression of a person’s emotions, the assessment and recognition of emotions in others, the regulation of a person’s emotions, and the use of emotion to aid performance at the workplace. The scale was developed based on the^46^ definition of EI.

In all, a 16-item questionnaire focusing on awareness of emotion in oneself; awareness of emotion in others; use of emotions; and emotion control were used. Table 2 below presents the different dimensions and definitions of EI based on the WLEIS.

**Table 2:** Dimensions for EI.

EI dimension	Definition
Self-emotion appraisal (SEA)	Measures workers’ ability to understand and value their own emotions. It also assesses the worker’s capacity to comprehend their deepest emotions and how they express them naturally in the workplace
Others’ emotion appraisal (OEA)	Assesses Workers’ abilities to perceive and understand the emotions of people around them during work and task-related activities
Use of emotion (UOE)	Measures workers’ capacity to use their own emotions to route them towards constructive activities and personal performance at the workplace
Regulation of emotion (ROE)	Assesses workers’ ability to regulate their emotions, which empowers them to recover more quickly from mood swings and anxiety during work

#### The safety performance construct

Safety performance refers to changes in employee work behaviours expected to prevent or reduce workplace accidents, occupational injuries, and illness. Measuring safety performance is not direct as safety variables among organizations also differ. In most cases, some organizations adopt injury or accident logs to determine workers’ safety performance. This study, however, adopted the construct of^27^ which described safety performance as the level of safety compliance and safety participation among workers^89^. While safety compliance refers to the level of employees’ adherence to safety rules, procedures, regulations, and standards related to their work, safety participation includes the level of employees’ involvement in safety programs, safety meetings, and helping others at work to adhere to safety standards^23^. Both dimensions of measuring safety performance focus on employee behaviours geared towards accident and injury prevention at the workplace. Therefore, a 7-item questionnaire adapted from the study of^23^ was used to measure respondents’ safety participation and safety compliance level in the organization. The safety performance items were related to the respondents' own safety participation and safety compliance behaviour towards safety at work.

#### Accidents

In the oil and gas sector, the use of chemicals can be toxic, corrosive, flammable, and combustible, hence may pose various degrees of work-related hazards or risks to workers. Chemical hazards can cause acute harm, such as burns, irritation and vomiting, or create chronic health issues, such as asthma, liver damage and cancer. Again, physical hazards such as extreme temperatures, poor air quality, excessive noise and radiation in the workplace can all cause work-related injuries and accidents. In this study, workplace accidents refer to discrete occurrences in the course of work that may lead to fatal or non-fatal injuries to the employee^90^. This definition includes accidents occurring only at the workplace while the employee is actively engaged in an officially assigned task by the employer or superior. A 6-item questionnaire for workplace accidents adopted from the study of^91^ was used to assess the frequency of accidents encountered by the workers. The Questionnaire was slightly modified in order to fit the measurement Scale of the accident variable.

#### Scale

Variable items for OHSMP, safety participation, and EI were measured on a 5-point Likert Scale ranging from strongly agree to strongly disagree. Each statement for OHSMP (e.g., “Safety officers and safety supervisors carry out safety inspections at regular intervals to detect hazards”), safety performance (i.e., I ensure the highest levels of safety when I carry out my job) and EI (i.e., I always know my friends’ emotions from their behaviours) were all coded as 1 = Strongly Disagree and 5 = Strongly agree. The response values for safety compliance were reverse-coded. Items for workplace accidents were ranked from very often to not very often (e.g., I have experienced some level of burns in my line of duty) and were coded as 1 = not very often and 5 = very often. The study considered reported work accidents that occurred only in the last 12 months.

### Procedure for data analysis

The study followed a strict and robust quantitative analysis to assess the data. Descriptive and reliability analysis was initially performed to ascertain the means, standard deviation, and Cronbach’s alpha of all scales. The Kaiser–Meyer–Olkin Measure of Sampling Adequacy (KMO-MSA) and Bartlett’s Test of Sphericity (BTS) was also estimated. Consistent with prior works of^92^, the study set a strict significance level for the regression coefficients at (95%) for each latent variable and accordingly evaluated the reliability, validity, and internal consistency of each latent variable. Fit indices such as the Chi-square, Root Mean Square Error of Approximation (RMSEA), Comparative Fit Index (CFI), Standardised Root Mean Residual (SRMR), Tucker Lewis index (TLI), and Goodness of Fit (GFI) were closely monitored to establish a good fit. Further, the procedures of Baron and Kenny’s^93^ bootstrapping method, which assesses the indirect effect of the mediating variable, were followed to estimate the mediating effect of EI. The Statistical Product and Service Solutions (SPSS) version 26.0 (IBM, Ar-monk, NY, USA) was used for data coding, descriptive, reliability and validity analysis. In contrast, the confirmatory factor analysis was all estimated using version 25.0 of Analysis of a Moment Structures (AMOS).

### Ethical approval

The study was approved by the School of Management of the Jiangsu University Research and Ethical Committee headed by Prof. Du. Informed consent was also obtained from all organizations and individual participants included in the study.

### Informed consent

Informed consent was obtained from all subjects involved in the study.

## Results

### Participants demographics

As earlier stated, this study was conducted with the voluntary collaboration of 699 oil and gas workers. The majority of these respondents were males, representing 72.9%. Most of the participants as well are between the ages 20–40 years (75%), married (67.09%), and of the Christian religion (75.67%). Again, more participants hold graduate degree (55.5%), and most with work experience between 6 and 15 years (57.8%). The sample-specific characterization is presented in Table 3.

**Table 3:** Demographics of respondents.

Variable	Characteristics	Frequency	Percentage
Gender	Male	507	72.93
	Female	192	27.06
Age	20–30 years	245	28.66
	31–40 years	296	47.46
	41–50 years	131	17.46
	51 years and above	27	6.40
Marital status	Never married	204	29.18
	Married	469	67.09
	Divorced	26	3.71
Religion	Christianity	529	75.67
	Islamic	158	22.60
Education	NVTI/Technical/SHS Certificate	109	14.53
	HND/Degree	364	55.33
	Postgraduate	101	13.46
	Professional	96	12.80
	Others	29	3.86
Working experience	1–5 years	199	26.53
	6–10 years	261	41.60
	11–15 years	122	16.26
	16–20 years	65	8.66
	21 years and above	52	6.93
Type of work	Engineers	88	12.58
	Maintenance personnel	211	30.18
	Labourers	127	18.16
	Technicians	99	14.16
	Machine operators	174	24.89

### Factor loading and descriptive analysis

The results as presented in Table 4 shows the mean, standard deviation and factor loadings for items used to measure the constructs. The Kaiser–Meyer–Olkin Measure of Sampling Adequacy (KMO-MSA) and Bartlett’s Test of Sphericity (BTS) are also presented. The results show that the factor loadings for all items were above 0.700. On the other hand, the results for the KMO-MSA estimates for the Scales were all above the 0.600 threshold while BTS for the Scales were as well significant.

**Table 4:** Factor loadings and descriptive statistics.

Measurement variable	Factor loadings	Means	SD	KMO-MSA	BTS Sig
Occupational Health and Safety Management Practices (OHSMP)				**0.873**	**0.000**
Inspection of Organizational Hazards (IOH)				**0.767**	**0.000**
IOH1: Safety officers and safety supervisors carry out safety inspections at regular intervals to detect hazards	0.888	1.951	0.959		
IOH2: There exist appropriate arrangements to ensure that actions are taken because of the findings of safety inspections	0.897	2.022	0.912		
IOH3: There exist appropriate arrangements to monitor the effectiveness and thoroughness of eliminating hazards after inspection	0.891	2.011	0.951		
IOH4: There exist appropriate arrangements to collate and analyse the results of safety inspections after hazard elimination	0.914	1.982	0.902		
Safety Policies, Procedures and Standards (SPS)				**0.779**	**0.000**
SPS1: The safety policies commit the organization to full compliance with all relevant health and safety legislation	0.901	2.483	0.928		
SPS2: The policy set targets for health and safety performance including a commitment to progressive improvement	0.902	2.585	0.912		
SPS3: Safety policies and procedures are explained to new employees as part of their training and orientation before entry to and working on-site	0.904	2.543	0.943		
SPS4: There exist effective arrangements for reviewing the health and safety policy at least once a year	0.911	1.615	0.911		
Personal Protection Program (PPP)				**0.872**	**0.000**
PPE1: All plants and equipment used on site are suitable for the job and fit for the purpose	0.879	1.526	0.905		
PPE2: Safety policies exist on the proper use of plants and machines at the worksite	0.885	2.47	0.947		
PPE3: There exist procedures and instructions to ensure the proper use of PPEs	0.790	2.417	0.968		
PPE4: There is a sufficient stock of carefully selected and appropriate PPE at all times	0.760	1.376	0.806		
Safety Training (ST)				**0.888**	**0.000**
ST1: My company gives comprehensive training to the employees in workplace health and safety issues	0.877	2.457	1.011		
ST2: Newly recruits are trained adequately to learn safety rules and procedures	0.877	2.516	0.983		
ST3: The safety training given to me is adequate to enable me to assess hazards in the workplace	0.873	2.487	0.967		
ST4: Safety issues are given high priority in training programmes	0.872	2.426	0.973		
Management Commitment to Safety (MC)				**0.709**	**0.000**
MC1: Management provides safety information	0.887	4.176	1.069		
MC2: Management conducts frequent safety inspections	0.859	4.076	1.053		
MC3: Management rewards safety workers	0.884	3.478	1.058		
MC4: Management always provides adequate safety information	0.923	4.398	1.078		
Safety Performance (SP)				**0.896**	**0.000**
Safety Compliance (SP)				**0.801**	**0.000**
SP1: I know how to perform my job in a safe manner	0.922	2.421	0.591		
SP2: I use all necessary safety equipment to do my job	0.811	3.012	0.682		
SP3: I know how to reduce the risk of accidents and incidents in the workplace	0.901	2.112	0.652		
SP4: I know what the hazards are associated with my job and the necessary precautions to be taken while doing my job	0.879	2.003	0.742		
Safety Participation (SPA)				**0.991**	**0.000**
SPA1: I put extra effort to improve the safety of the workplace	0.808	3.001	0.591		
SPA2: I voluntarily carry out tasks or activities that help to improve workplace safety	0.766	3.012	0.682		
SPA3: I ensure the highest levels of safety when I carry out my job	0.898	3.112	0.652		
SPA4: I encourage my co-workers to work safely	0.877	2.993	0.742		
Workplace Accidents (WPA)				**0.829**	**0.000**
WPA1: Over the past 6 months to 1 year I have been struck against something fixed or stationary	0.891	3.011	0.919		
WPA2: Over the past 6 months to 1 year I have Overextended myself lifting or moving things	0.901	3.962	0.812		
WPA3: Over the past 6 months to one 1 year I have been exposed to chemicals such as gases and fumes	0.933	4.114	0.911		
WPA4: Over the past 6 months to one 1 year I have fallen off something (e.g., a ladder, shelf, etc.)	0.921	3.735	0.922		
WPA5: Over the past 6 months to 1 year I have been trapped by something collapsing, caving in or overturning	0.891	2.776	0.933		
WPA6: Over the past 12 months to one 1 year I have slipped and fallen whiles going about my work duties	0.877	3.111	1.721		
Emotional Intelligence (EI)				**0.777**	**0.000**
Self-Emotion Appraisal (SEA)				**0.678**	**0.000**
SEA1: I have a good sense of why I have certain feelings most of the time	0.833	3.014	0.911		
SEA2: I have a good understanding of my own emotions	0.821	2.935	0.922		
SEA3: I understand what I feel	0.991	2.006	0.933		
SEA4: I always know whether or not I am happy	0.801	1.911	1.721		
Others’ Emotion Appraisal (OEA)				**0.711**	**0.000**
OEA 1: I always know my friends’ emotions from their behaviour	0.813	2.444	0.911		
OEA 2: I am a good observer of others’ emotions	0.811	2.035	0.922		
OEA 3: I am sensitive to the feelings and emotions of others	0.801	3.007	0.933		
OEA 4: I have a good understanding of the emotions of people around me	0.911	1.999	1.721		
Use of Emotion (UOE)				**0.809**	**0.000**
UOE 1: I always set goals for myself and then try my best to achieve them	0.888	2.110	0.912		
UOE 2: I always tell myself I am a competent person	0.824	4.081	0.978		
UOE 3: I am a self-motivated person	0.836	3.019	0.982		
UOE 4: I would always encourage myself to try my best	0.854	3.043	0.943		
Control of Emotion (COE)				**0.699**	**0.000**
COE 1: I am able to control my temper and handle difficulties rationally	0.919	4.114	0.911		
COE 2: I am quite capable of controlling my own emotions	0.867	3.735	0.922		
COE 3: I can always calm down quickly when I am very angry	0.809	2.776	0.933		
COE 4: I have good control of my own emotions	0.807	3.111	1.721		

Significant values are in bold.

### Reliabilities and convergence validity analysis

The results for the AVE and CR for all constructs as presented in Table 5 satisfies all conditions of a convergent validity test. Thus, the AVE and CR were above the threshold of 0.5 and 0.7, respectively. The Cronbach’s alpha for all constructs as well were all above 0.700. These results show that each scale for measuring the constructs has met the needed reliability and validity criteria.

**Table 5:** Reliabilities and convergence validity.

Construct	Cronbach alpha	Average variance extracted	Composite reliability
OHSMP	**0.801**	**0.795**	**0.936**
Organizational Hazards (OH)	0.778	0.720	0.911
Safety policies, procedures and standards (SPS)	0.818	0.690	0.898
Plant and equipment’s/personal protection equipment (PPE)	0.709	0.693	0.900
Safety training (ST)	0.921	0.601	0.859
Management commitment to safety (MC)	0.779	0.667	0.888
Safety performance (SP)	**0.799**	**0.665**	**0.855**
Safety compliance	0.644	0.644	0.843
Safety participation	0.791	0.512	0.922
Workplace accidents (WPA)	**0.723**	**0.702**	**0.933**
Emotional intelligence (EI)	**0.744**	**0.737**	**0.846**
Self-emotion appraisal (SEA)	0.801	0.708	0.905
Others’ emotion appraisal (OEA)	0.709	0.540	0.778
Use of emzotion (UOE)	0.701	0.645	0.878
Control of emotion (COE)	0.764	0.587	0.810

Significant values are in bold.

### Discriminant validity test

Because each scale's square root of AVE was higher than its association with other scales, the results shown in Table 6 demonstrate that the scales are separate and unrelated. Simply put, this demonstrates that the items are distinct and do not prejudice their measurement construct^94^.

**Table 6:** Discriminant validity test.

Variables	OHSMP	SP	WPA	EI	SEA	UOE	OEA	COE
Occupational Health and Safety Management Practices–OHSMP	**(0.820)**							
Safety Performance-SP	− 0.375	**(0.823)**						
Workplace Accidents-WPA	− 0.501	− 0.441	**(0.837)**					
1 Emotional intelligence-EI	0.284	0.468	− 0.421	**(0.792)**				
Self-emotion appraisal (SEA)	0.287	0.501	− 0.483	0.579	**(0.841)**			
Use of emotion (UOE)	0.246	0.481	− 0.554	0.447	0.536	**(0.735)**		
Others’ emotion appraisal (OEA)	0.317	0.466	− 0.275	0.368	0.341	0.409	**(0.803)**	
Regulation of emotions (ROE)	0.239	0.374	− 0.227	0.374	0.383	0.294	0.247	**(0.766)**

Correlations above 0.3 were all significant at *p < 0.05 (Interpretation of results was based on 2 decimals). Figures in the bracket represent the discriminant validity estimates for the factors.

### Confirmatory factor analysis (CFA)

The study reported the model fit indices for the confirmatory analysis results to confirm if the items adopted for measuring the constructs fit their purpose. The values were Chi-square, Goodness of Fit Index (GFI), Root Mean Square Error Approximation (RMSEA), Comparative Fit Index (CFI), Tucker–Lewis Index (TLI), and The Standardized Root Mean Square Residual (SRMR). The fit indices’ results show that all the constructs meet the threshold for the fit criterion. Table 7 presents the model fit indices for the CFA of the Scales.

**Table 7:** Model fit indices for the CFA.

Constructs	${\chi }^{2}/df$	GFI	SRMR	RMSEA	CFI	TLI
Occupational Health and Safety Management Practices (OHSMP)	4.393	0.951	0.031	0.048	0.979	0.957
Safety performance (SP)	3.666	0.937	0.037	0.045	0.911	0.903
Workplace accidents (WPA)	4.721	0.829	0.041	0.031	0.877	0.821
Emotional intelligence (EI)	4.987	0.922	0.048	0.041	0.912	0.899

### The standard multiple regressions results

The standard multiple regression results indicated in Table 8 reported the regression estimates, R-squared statistics, F-statistics, and T-statistics to assess the significance of the causal relationship among the constructs.

**Table 8:** Standard multiple regression results.

Variables	*Β*	*R*^*2*^	*F*	*T*
OHSMP and Safety Performance (SP)	− 0.375	0.312	25.051*	− 4.371*
OHSMP and Workplace Accidents (WPA)	− 0.501	0.521	59.059*	− 7.011*
OHSMP and EI	0.284	0.234	16.557*	4.429**
OHSMP and self-emotion appraisal (SEA)	0.287	0.250	12.672**	4.555**
OHSMP and use of emotion (UOE)	0.246	0.211	9.899**	4.302**
OHSMP and others’ emotion appraisal (OEA)	0.317	0.272	20.317**	4.072**
OHSMP and control of emotion (COE)	0.239	0.229	9.001*	4.222*
Safety performance (SP) and workplace accidents (WPA)	− 0.441	0.503	49.169**	− 6.093**
EI and safety performance (SP)	0.468	0.338	26.117*	5.437*
Safety performance and self-emotion appraisal (SEA)	0.501	0.409	31.129**	6.233**
Safety performance and use of emotion (UOE)	0.481	0.397	29.039**	5.102**
Safety Performance and others’ emotion appraisal (OEA)	0.466	0.353	26.715*	5.711*
Safety Performance and control of emotion (COE)	0.374	0.339	25.031*	4.891*
EI and workplace accidents (WPA)	− 0.421	0.322	23.097*	− 4.099*
Workplace accidents and self-emotion appraisal (SEA)	− 0.483	0.450	33.719**	− 6.549**
Workplace accidents and use of emotion (UOE)	− 0.554	0.432	31.227*	− 7.377*
Workplace accidents and others’ emotion appraisal (OEA)	− 0.275	0.237	11.479*	− 4.222*
Workplace accidents and control of emotion (COE)	− 0.227	0.179	5.991**	− 4.002**

*p < 0.01, **p < 0.05*.*

The results, as reported by the standard multiple regressions, show that OHSMP significantly predicts WPA (β = − 0.501), SP (β = − 0.375), and EI (β = 0.284). The results also indicated that EI significantly determines SP (β = 0.468) and WPA (β = − 0.411), while SP significantly predicts WPA (β = − 0.441). The F and T estimate in the regression table also confirms a significant relationship between these variables.

The R^2^, which measures the coefficient of determination, also shows that OHSMP may determine 52.1% (R^2^ = 0.521) of the variation in WPA, 31.2% (R^2^ = 0.312) of the variation in SP and 23.4% (R^2^ = 0.234) of the variation in EI. The R^2^ further showed that EI may also account for 33.8% (R^2^ = 0.338) of the variation in SP and 32.2% (R^2^ = 0.322) of the variations in WPA. More specifically, SEA is likely to account for the highest variation of determination in SP and WPA than any other form of EI. Thus, the results show that SEA is likely to determine about 40.9% (R^2^ = 0.409) of the variation in SP and 45.0% (R^2^ = 0.450) in WPA. Similarly, OHSM may account for 25.0% (R^2^ = 0.250) of the variations in SEA, 21.1% (R^2^ = 0.211) of the variation in UOE, 27.2% (R^2^ = 0.272) of the variation in OEA and 22.9% (R^2^ = 0.229) of the variation in COE. Lastly, SP probably accounted for 50.3% (R^2^ = 0.503) of the variations in WPA.

### Results of mediation analysis

In assessing the mediation effect, the^95^ bootstrapping estimation procedure was adopted. The study further used bias-corrected and accelerated bootstrapping to establish confidence intervals (CIs)^96^. Sobel^97^ suggested that to identify a full or partial mediation, the reduction in variance explained by the independent variable must be significant, as determined by numerous tests. The study, therefore, adopted the estimation of the variation accounted for (VAF) as proposed by^94^ to determine the existence of significance of the mediation. According to Hair et al.^94^, the VAF determines the degree to which the direct path has been absorbed. Hence, a VAF below 0.20 indicates no mediation, while a VAF ranging from 0.20 to 0.80 denotes partial mediation. Finally, when the VAF value is above 0.80, a full mediation exists. Tables 9 and 10 present the mediation analysis results by presenting the total, direct, and indirect effects and their 95% CIs.

**Table 9:** Results of the mediating analysis (OHSMP–EI–SP).

	Estimate	95% CI Lower	95% CI Upper	*p*-value
OHSMP–EI–SP				
Direct	− 0.3750	− 0.1296	0.0392	0.0019
Indirect	0.1330	0.1272	0.0599	0.0031
Total Effect	− 0.2420	− 0.2673	0.6895	0.0123
VAF	0.2863			
OHSMP–SEA–SP				
Direct	− 0.3750	− 0.3356	0.0321	0.0041
Indirect	0.1440	0.1071	0.0233	0.0055
Total Effect	− 0.2310	− 2.3386	0.0619	0.0111
VAF	0.3213			
OHSMP–UOE–SP				
Direct	− 0.3750	− 0.3368	0.0199	0.0068
Indirect	0.1183	0.0771	0.1292	0.0077
Total Effect	− 0.2567	− 0.2338	0.1491	0.0101
VAF	0.2993			
OHSMP–OEA–SP				
Direct	− 0.3750	− 0.3413	0.0294	0.0073
Indirect	0.1477	0.0973	0.1798	0.0092
Total Effect	− 0.2273	− 0.2718	0.1993	0.0201
VAF	0.2719			
OHSMP–COE–SP				
Direct	− 0.3750	− 0.3441	0.0075	0.0019
Indirect	0.0894	0.0771	0.1097	0.0012
Total Effect	− 0.2856	− 0.2773	0.1183	0.0091
VAF	0.2414			

WPA, Workplace Accidents; EI, Emotional Intelligence; OHSMP, Occupational Health and Safety Management Framework, SEA, Self-Emotion Appraisal; UOE, Use of Emotion; OEA, Others’ Emotion Appraisal; COE, Control of Emotion.

**Table 10:** Results of the mediating analysis (OHSMPEI–WPA).

	Estimate	95% CI lower	95% CI upper	*p*-value
OHSMP–EI–WPA				
Direct	− 0.5012	− 0.6336	0.0784	0.0000
Indirect	− 0.1196	− 0.0952	0.0419	0.0001
Total EFFECT	− 0.6208	− 0.7253	0.0792	0.0031
VAF	0.2804			
OHSMP–SEA–WPA				
Direct	− 0.5012	− 0.6345	0.0410	0.0061
Indirect	− 0.1386	− 0.1962	0.0188	0.0035
Total effect	− 0.6398	− 0.8363	0.0611	0.0062
VAF	0.3001			
OHSMP–UOE–WPA				
Direct	− 0.5012	− 0.6356	0.0169	0.0033
Indirect	− 0.1362	− 0.0962	0.0272	0.0067
Total effect	− 0.6374	− 0.7328	0.0474	0.0131
VAF	0.2944			
OHSMP–OEA–WPA				
Direct	− 0.5012	− 0.6313	0.0273	0.0064
Indirect	− 0.0872	− 0.0763	0.0711	0.0069
Total effect	− 0.5884	− 0.7188	0.0999	0.0215
VAF	0.2766			
OHSMP–COE–WPA				
Direct	− 0.5012	− 0.6436	0.0088	0.0065
Indirect	− 0.0543	− 0.0462	0.0604	0.0089
Total effect	− 0.5556	− 0.6819	0.0577	0.0111
VAF	0.2444			

WPA, Workplace Accidents; EI, Emotional Intelligence; OHSMP, Occupational Health and Safety Management Framework, SEA, Self-Emotion Appraisal; UOE, Use of Emotion; OEA, Others’ Emotion Appraisal; COE, Control of Emotion.

From Table 7 above, the influence of OHSMP on SP without the mediation of EI indicated a significant effect of − 0.375, while the indirect effect of EI on the causal link between OHSMP and SP showed a significant effect of 0.133. The VAF value also showed that the indirect effect of EI explains 28.63% of the total effect of OHSMP on SP. This suggests that the effect of OHSMP on SP is partially mediated by EI (0.20 < VAF < 0.80). Similarly, the VAF value and the indirect effect of SEA, UOE, OEA, and COE as well indicated a partial mediation on the causal relationship between OHSMP and SP.

Concerning the mediation effect of EI in the causal relationship between OHSMP and WPA, the results presented in Table 8 showed an indirect effect of − 0.1196 for EI and a VAF of 28.04%. This also denotes a partial mediation and shows that the total effect of OHSMP on WPA is explained by the indirect effect of EI, and 28.04% of the influence OHSMP has on WPA is explained by the variations in EI. Likewise, the indirect effect and the VAF for all other dimensions of EI, thus, SEA, UOE, OEA, and COE, showed a significant partial mediation in the relationship between OHSMP and WPA.

**Figure 2:**
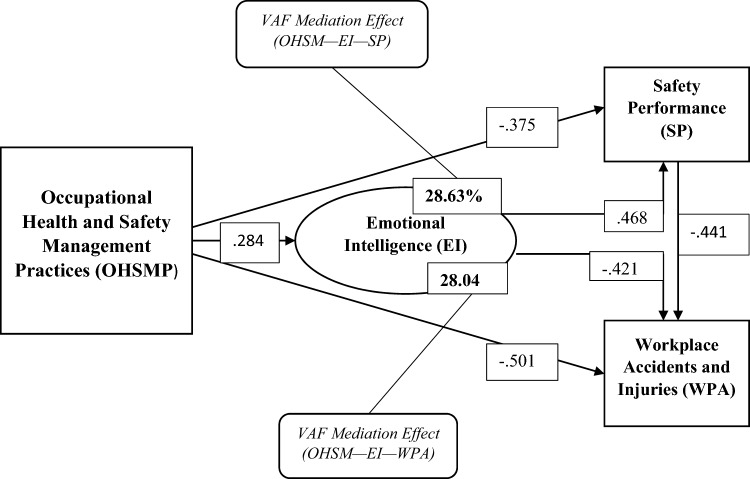
Causal relationship between variables.

The study further presented the findings of the research objectives graphically in Fig. 2 below:

## Discussions

Undoubtedly, global statistics on workplace accidents and injuries among industries continue to grow^98^. Specifically, industries in developing countries continue to record high fatalities and occupational injuries compared to developed countries^99^. Ghana falls among these developing countries with the highest estimated accident rate^4^. While several studies have highlighted the role of EI in the safety context^38^, this is the first study that addresses the mediation effect of EI in the influence of Occupational Health and Safety Management Practices (OHSMP) on safety performance and workplace accidents. Arguably, the literature available on how and the extent to which EI significantly promotes safety performance or safety outcomes remains limited and perhaps inconsistent. In line with this, the emotional contagion theory^100^, which suggested that emotional exchanges among workers can impact the flow of cognitive functioning and behaviours in their daily life, was adopted to demonstrate the mediation effect of EI in the impact OHSMP has on safety performance and workplace accidents.

The results suggest that OHSMP significantly and positively predicts EI but negatively predicts safety performance and workplace accidents. The negative relationship reported between OHSMP, and safety performance differs from most previous studies. The rising interest and investment in workplace health promotion raise no questions as a cost–benefit analysis of the subject matter. As put forward by most studies, investment in safety systems is likely to improve safety-related outcomes; however, the opposite is also true. Like most industries in Ghana, the oil and gas industry has yet to receive the needed occupational health attention despite being faced with numerous hazardous exposures. As reported by a few studies, lapses in the effective administration of occupational health and safety policies include the lack of safety policy directions, poor safety infrastructure, lack of funding for safety systems, a large number of unqualified occupational health professionals, inadequate OHS work-related accidents or injuries monitoring mechanism remain a challenge for most industries in Ghana^29–31,101^. Surprisingly as it may seem, Ghana as a country, despite the ginormous investments that the country attracts with its associated OHS-related issues, has yet to have a national policy on Occupational Health and Safety Standards for industries. Both past and present governments of Ghana have yet to invest any political will, commitment, and support for standardized and globally recognized occupational health and safety policies. Evidently, out of over 70 conventions of the ILO, only ten have been ratified by the government of Ghana, while the four core conventions on occupational health and safety are yet to be ratified. These and many other challenges have made most industries adamant about investing time, money, and commitment to promoting effective OHSM practices. Perhaps the need for promoting work safety has been sacrificed for profitability, which may account for the negative relationship between OHSMP and SP reported in this study. These challenges may also contribute to the negative association between OHSMP and workplace accidents.

On the other hand, EI was found to predict safety performance and workplace accidents significantly. The Baron and Kenny^95^ bootstrapping estimation procedure for the mediation effect of EI and the VAF results indicated that all the construct dimension for measuring EI (i.e., SEA, OUE, OEA, and COE) significantly explains the relationship between OHSMP and safety performance as well as the influence of OHSMP on workplace accidents. To put it more clearly, workers’ self-emotional appraisal, the use of emotions at work, the appraisal of others’ emotions and control of personal emotions are significant factors that determine safety performance and accidents encountered at the workplace. The findings of the study have also demonstrated that safety performance cannot only be achieved through the provision of effective and efficient health and safety management systems or practices, but it also involves the ability of safety leaders or the organization at large to move beyond the assessment of safety controls or understanding risk management. This is why Jeffries^4^ suggested that organizations must go beyond previous and current initiatives and rather address the individual workers’ attitudes and emotions as they relate to the working conditions, workers’ safety and the safety of others in order to a better safety outcome. This means that workers’ EI can aid in determining the vulnerability towards unsafe behaviours, which could derail the organization’s intentions or purposes of implementing specific OHSM practices or systems. Thus, organizations need to understand and comprehend how EI interacts with safety-related activities to help resolve minor related hazard issues, which may not even be solved through excessive safety or technical training.

Unlike plants and equipment, EI makes a significant difference in relation to how the employee tackles work and responds to organizational policies. As already highlighted, the pace of work activities in the organization can lead to emotional stress which can likely lead to the employee committing several mistakes including flouting safety management rules and standards and consequently being involved in work-related accidents. In most cases, when employees are expected to meet high production targets, they are likely to trade logic for emotions due to the fear of losing their jobs if they fail. This scenario is a suitable example to reiterate that EI interacts with OHSMP to influence safety performance and predict work-related workplace accidents. As opined by Ifelebuegu^15^ EI success factors such as ‘being able to rule one’s own emotions to facilitate thinking, ‘being able to deal with the emotions of others and ‘being able to discuss one’s own emotions accurately’ significantly determine the health and safety performance of workers.

Blakey and Abramowitz^28^ described safety performance as an immediate and contextual attitude towards preventing and minimising work-related hazards to improve work engagements. Therefore, workers who are more cautious and exhibit positive preventive attitudes are likelier to exhibit positive work behaviours. Putting the relationship between EI and safety performance in perspective, high EI may be good for work safety behaviour. Thus, people with high EI are inclined to control their emotions and behaviours, participate in safety decision-making, and will be able to report potential risks. Thus, they will help reduce work-related risks and potential accidents^102–104^. As described by Mayer and Salovey^46^, emotional perception, emotional assimilation, emotional understanding, and emotion management remain contributing factors that influence human attitudes, behaviours, job satisfaction and engagements at work^37,63^. This conforms to the theory of planned behaviour by Ajzen^105^ which identified perceived behavioural control and subjective norms as a major influence on a person’s behaviour at work. Similarly, Jeffries^14^ stated that safety-related behaviours are both participatory and compliance-based; hence the subjective norms and the need to conform to rules and policies are extrinsic reasons for compliance with safety requirements. Thus, it is expected that more altruistic people, as opposed to egocentric, will be more likely to demonstrate safe work behaviours due to the concern they extend to others before acting. This is also supported by the study of Abraham^106^, who demonstrated that people who exhibit high EI are more cooperative toward task performance and more likely to exhibit self-control by demonstrating a thought process that will result in decisions that support the promotion of a safe work environment^14^.

Petitta et al.^35^ further argued that workers who exhibit emotional anger are likely to experience cognitive failures at work, whereas those with joy do not. Their study further suggested a strong significant relationship between accident experience and cognitive failures at work. Thus, if workers fail to exhibit EI during social interaction at work but rather show negative emotions like anger, the probability of experiencing workplace accidents is expected to be high. Their findings confirm the results of this current study, which also suggest that EI significantly determines accidents encountered at the workplace. The problem of dealing with workplace accidents is, therefore, likely to be embedded in workers’ inability to exhibit positive EI during interaction with people at the workplace or dealing with factors such as depression, anxiety, or burnout while performing various tasks. In line with the Affective Primacy Theory (APT)^107–109^, this analogy is also likely to explain the significant influence of EI on safety performance, as this current study found safety performance to predict accidents encountered at the workplace significantly. As suggested in previous literature, encouraging employees to practice safe work behaviour will likely reduce workplace accidents and occupational injuries^110,111^.

As reported by the Occupational Health and Safety Administration (OSHA), a single fatality that occurs at work is likely to cost up to $910,000, while a prevented workplace accident that can potentially cause injury saves the organization $28,000^112^. Geller and Wiegand^113^ also proposed that creating a safer work environment must specifically focus on the work environment itself, the people involved in the work, and their behaviour. For instance, Jamal and Khan^114^ identified ignorant behaviour and attitude from both employers and employees as significant predictors of behavioural safety and non-compliance to health and safety requirements. Whatever the case may be, the burden of providing and promoting a conducive and safe work environment lies on the shoulders of employers. It is also the responsibility of employees to work in accordance with all safety procedures, principles, and standards.

To conclude, it is important to iterate that the oil and gas industry must incorporate or integrate the development of EI (i.e., the ability to control personal emotions and the ability to deal with others’ emotions) into their regular human resource and safety management promotion programmes. As posited by Khandan and Vosughi^115^, workers who undergo emotional capability training are more productive and less likely to encounter workplace accidents. Similarly, Jeffries^14^ opined that providing employee training influences their perceived behavioural control. Practical safety training has also been used as an effective tool to educate workers on the prevention of potential work-related risks and workplace accidents. Hence, it enhances safety awareness and positively influences employees’ behaviour^88,116^. Additionally, Wang, Jiang, and Blackman^16^ added that EI is significantly related to situational awareness at the workplace only when safety training inadequacy was more salient. Thus, the more inadequate safety training was, the greater the negative indirect effect of EI on safety performance via situational awareness was, suggesting that effective safety training may complement employees’ low EI in shaping their situational awareness and safety behaviours. Organizations must, therefore, extend serious attention toward safety-related EI training and the development of workers’ potential to enhance positive emotional interaction at work. Workers can learn how to manage their emotions effectively even when under pressure or exposed to a hazardous work environment, as long as they are shown how to do so. This is what it means to use EI in safe environments. It is expected that such initiatives could lead to the improvement in safe work behaviours and safety performance.

## Conclusions

Employees’ health and safety at the workplace are of psychological and emotional concern to the worker as it is directly linked to their quality of life. Unlike previous studies, this current study theoretically and empirically provided an in-depth understanding of the significant intervention effect of four-dimensional constructs of EI in the causal link between OHSMP and safety performance and workplace accidents. The findings of the study have shown that all dimensions of EI are significant factors for decreasing workplace accidents and enhancing safety performance. The theoretical basis for these findings is that workers with high-level EI are likely to be able to cope with occupational health and safety lapses or safety-related challenges at the workplace by participating and complying with the organization’s safety management practices or procedures. Such employees are likely to exhibit safe working behaviours and contribute to improving safety performance in the organization. As suggested by Kim et al.^117^, when facing a disadvantaged situation, people with higher levels of EI are more likely to take proactive actions than passive ones. Hence, there is a need for organizations to divert attention towards the promotion of emotional training and development programs. The findings call for immediate intervention from industry players, specifically those in the oil and gas sector, to focus on developing programs to improve safety performance through positive emotional attitudes and behaviours.

### Limitations

Although this study contributes more to the previous literature, its findings also warrant further investigation. The issue of measuring people’s EI is quite complex. The study is, therefore, subjected to limitations such as socially desirable and modal responses due to the self-report measures adopted for data collection. Responses to workplace accidents, for instance, are expected to be subjected to some level of bias. As asserted by Lusk et al.^118^, self-report measures of accidents are related to independent observations of social desirability**.**

Again, the dependent and independent variables used for the study were slightly modified to fit the intent of what was being measured; hence, the analysis is prone to common method bias. Future studies should, therefore, consider investigating using other data methods, such as behavioural observation methods. There is some disparity among the sample groups who participated in the survey. Hence, the findings are likely to face some basic bias in responses. Apart from EI, other mediating factors such as skills and motivation, work pressure, psychological distress, and burnout can contribute to the influence of OHSMP on safety performance and workplace accidents. Future studies can consider multiple mediators. Other dimensions of EI, like the exhibition of fear or anxiety, while working in hazardous industries, are also likely to determine safety performance and predict workplace accidents. Therefore, future studies can examine the influence of such variables.

## Data Availability

Data is available upon request from researchers who meet the eligibility criteria. Kindly contact the first author privately through e-mail.
